# Editorial: Diagnosis, prevention and treatment in diabetic nephropathy

**DOI:** 10.3389/fendo.2022.1011665

**Published:** 2022-09-01

**Authors:** Maria Margherita Rando, Martina Guthoff, Vinod Tiwari, Federico Biscetti

**Affiliations:** ^1^ Department of Cardiovascular Sciences, Cardiovascular Internal Medicine Unit, Fondazione Policlinico Universitario A. Gemelli IRCCS, Roma, Italy; ^2^ Department of Diabetology, Endocrinology, Nephrology, Section of Nephrology and Hypertension, University Hospital Tübingen, Tübingen, Germany; ^3^ Department of Pharmaceutical Engineering & Technology, Indian Institute of Technology (B.H.U), Varanasy, India

**Keywords:** diabetes mellitus, kidney, diabetic nephropathy, chronic kidney disease, end-stage kidney disease

Diabetic nephropathy (DN) is one of the microvascular complications of diabetes affecting 30-40% of diabetic patients and represents the leading cause of end-stage kidney disease (ESKD). Treatment strategies are rare. Given the significant healthcare impact and high economic burden of DN, there is an urgent need for adequate and targeted management of the disease for early diagnosis and prevention of progression to ESKD.

Articles of this Research Topic provide a general overview of DN, highlight the importance of early detection of this disease, and suggest new diagnostic tools and treatment strategies.

The magnitude of the problem has been well described by Deng and colleagues, showing that diabetes-related chronic kidney disease (CKD) represents the sixth-leading cause of disability and fourth-leading cause of death globally Deng et al. In addition, they show that the middle socio-demographic index (SDI) quintile regions were the most interested by DN in 2019, while China, the United States and India were the countries with the highest burden of diabetes-related CKD Deng et al. Specifically, in a Bayesan age-period-cohort analysis, Wu et al. show that DN deaths in China could be on the rise, with DN deaths projected to reach 88803 in 2030, a 223.2% increase from 1990. Therefore, comprehensive prevention, early diagnosis and development of new therapeutic strategies are critical to reduce DN progression and related mortality.

DN is clinically characterized by proteinuria, hypertension, and progressive deterioration of renal function. Its main pathological features are mesangial expansion to nodular accumulations, glomerular basement membrane thickening, glomerulosclerosis, tubular atrophy, interstitial inflammation and tubule-interstitial fibrosis.

Multiple factors are involved in the pathogenesis of DN, including a hyperglycemic environment, oxidative stress, inflammation, and fibrosis (Duan et al.). In particular, understanding the mechanisms underlying DN development is important to find new and specific biomarkers to make diagnosis, as shown in the review by Duan et al..

To date, glomerular injury is considered central to the pathogenesis of DN, and estimated glomerular filtration rate and albuminuria are, by far, the most commonly used parameters to assess renal function and DN progression. Recent evidence, however, focuses on the importance of renal tubular injury in determining reduced kidney function, even in the early stage of disease (Duan et al., Chang et al.). It has been shown that people with diabetes without proteinuria develop kidney disease (Chang et al.). Furthermore, in the absence of microalbuminuria, tubular plasma markers may also be associated with early renal injury (Duan et al.). Specifically, Duan et al. present in their review the latest evidences related to markers of renal tubular injury, including neutrophil gelatinase-associated lipocalin, kidney injury molecule 1, YKL-40, monocyte chemoattractant protein-1, cubilin and megalin. Another study by Lee et al. shows an association between tubulointerstitial injury in patients with DN and specific urinary tubulointerstitial mRNA biomarkers (LYZ, C3, FKBP5 and G6PC), and even assesses their predictive role in ESKD progression. This study, however, does not examine patients with early DN, so these results cannot be extrapolated to early kidney injury. The Research Topic also introduces other molecules as markers of renal damage. Indeed, in their study, Huang et al. show that higher urinary sodium excretion is associated with urinary albumin-to-creatinine ratio and DN risk, possibly through mechanisms dependent on vascular sclerosis or insulin resistance. No clear association between natriuresis and DN, however, has been found (Huang et al). Xu et al. analyze the role of specific lipid molecules in DN and find an association between lysophosphatidylethanolamine (LPE) and triacylglycerol (TAG) 54:2-FA18:1 and DN risk. Furthermore, they find that LPE, TAG 54:2-FA18:1 and phosphatidylethanolamine (PE) levels are associated with microalbuminuria (early DN) and macroalbuminuria (late DN), suggesting that these biomarkers can be used for early diagnosis of DN (Xu et al.). Han et al. present an innovative non-invasive diagnostic tool for diagnosing and predicting DN severity. Indeed, they assess the role of specific salivary glycopatterns in predicting DN progression. Another promising non-invasive diagnostic technique has been presented by Hu et al. in an animal model study that analyzes the role of magnetic resonance in the detection of preclinical DN, specifically through apparent diffusion coefficients and decreased fractional anisotropy techniques.

Several risk factors distinct from diabetes have been associated with the progression of DN, and some articles on this topic have been considered some of them, leading us to reflect on the importance of considering such components in DN prevention and possibly treatment.

In particular, a study by Yen et al. shows a strong correlation between diabetes and hypertension, suggesting that the coexistence of the two disorders is associated with a higher incidence of CKD. Furthermore, they show that diabetic patients who subsequently develop hypertension have a very high hazard ratio for end-stage-renal-disease compared with hypertensive patients who later develop diabetes. Along the same lines, in a metanalysis, Ren et al. show that patients with higher lipoprotein A levels have higher risk of developing DN. It is, however, unclear whether higher lipoprotein A levels are the result of abnormal renal metabolism due to loss kidney function or increased hepatic protein synthesis due to renal protein loss. Furthermore, it is unclear whether lipoprotein A may represent a marker of kidney injury or whether it is involved in the pathogenesis of DN through its atherogenic effects. CKD may also be exacerbated by acute kidney injury, which is a mortality risk factor for people with diabetes (Mo et al.). The effect of the gender has even been assessed in literature. In particular, multiple lines of evidence show an association between males and the risk of DN progression and death. Wang et al., however, are unable to find this association, and in a group of patients who underwent kidney biopsy, women have higher blood pressure, total cholesterol and LDL cholesterol compared with men, but a lower proportion of higher grades CKD histology.

Currently, clinical strategies to reduce DN progression are limited. Current treatment options include angiotensin-converting enzyme (ACE) inhibitors and angiotensin II receptor blockers (ARB). More recently, sodium-glucose cotransporter -2 (SGLT-2) inhibitors and novel non-steroidal mineral receptor antagonists have received attention for their anti-inflammatory and cardioprotective effects (Duan et al.). The meta-analysis by Li et al. describes the available evidence regarding the treatment of DN with tripterygium glycoside, a component of Chinese medicine with immunosuppressive effects. This work finds that tripterygium glycoside reduces serum and urinary biomarkers levels of DN progression, but at the risk of side effects. Another review by Wang et al. summarizes the results of various studies using mesenchymal stem cells and describes possible applications in DN therapy. More research, however, is needed to clarify the risks and benefits of these treatments (Li et al.).

Overall, all the articles of this Research Topic give a broad overview of new strategies for DN diagnosis, prevention and treatment, providing new insights and future perspective for research in the field.

## Author contributions

MR and FB wrote the article. MR, FB, MG and VT participated as guest editors for manuscripts of the Research Topic, where they were not coauthors themselves. All authors listed have made a substantial, direct and intellectual contribution to the work, and approved it for publication.

## Acknowledgments

MR, FB, MG, and VT thank all authors, reviewers, and external editors for their valuable contributions to this Research Topic.

## Conflict of interest

The authors declare that the research was conducted in the absence of any commercial or financial relationships that could be construed as a potential conflict of interest.

## Publisher’s note

All claims expressed in this article are solely those of the authors and do not necessarily represent those of their affiliated organizations, or those of the publisher, the editors and the reviewers. Any product that may be evaluated in this article, or claim that may be made by its manufacturer, is not guaranteed or endorsed by the publisher.

